# Management of Anticoagulation in Antiphospholipid Syndrome and Systemic Lupus Erythematosus With Concurrent Thrombosis and Hemorrhage

**DOI:** 10.7759/cureus.87122

**Published:** 2025-07-01

**Authors:** Teresa Mota, Rita Queirós Pereira, Diana Leão, Ana Maria Leite, Fernando Araújo

**Affiliations:** 1 Immunohemotherapy Department, Unidade Local de Saúde de São João, Porto, PRT

**Keywords:** antiphospholipid syndrome, diffuse alveolar hemorrhage, lupus flare, pulmonary-renal syndrome, systemic lupus erythematosus, therapeutic anticoagulation

## Abstract

Antiphospholipid syndrome (APS) is an autoimmune disorder characterized by arterial, venous, or small-vessel thromboembolic events and/or pregnancy morbidity in the presence of antiphospholipid antibodies. APS can occur in the setting of an underlying systemic autoimmune disease, particularly systemic lupus erythematosus (SLE). SLE is a chronic autoimmune disease that can affect virtually any organ of the body due to the production of antinuclear antibodies. During the course of their disease, many patients develop pulmonary manifestations, including alveolar hemorrhage.

We describe a clinical case of a 35-year-old woman with APS and SLE, anticoagulated with warfarin, who went to the emergency room with asthenia and productive cough with hemoptysis, and was diagnosed with a lupus flare and pulmonary-renal syndrome. Anticoagulation had to be discontinued due to the high risk of recurrent bleeding (HAS-BLED score 3). This case report describes the difficulty in managing the anticoagulant therapy in a patient with APS and SLE with both thrombotic and hemorrhagic manifestations.

## Introduction

Antiphospholipid syndrome (APS) is an autoimmune disorder characterized by arterial, venous, or small-vessel thromboembolic events and/or pregnancy morbidity in the presence of persistent antiphospholipid antibodies (aPLs) [1]. In a large retrospective analysis including patients without known autoimmune diseases, aPLs were present in approximately 6% of patients with pregnancy morbidity, 13.5% with stroke, 11% with myocardial infarction, and 9.5% with deep vein thrombosis (DVT) [2].

Approximately half of patients with APS have the primary form of the disease, while the other half have an associated systemic autoimmune disorder. Systemic lupus erythematosus (SLE) is the most commonly linked condition, occurring in about 35% of cases [3].

SLE is a chronic autoimmune disease that can affect virtually any organ of the body due to the production of antinuclear antibodies (ANAs). During the course of their disease, many patients develop pulmonary manifestations including alveolar hemorrhage. Diffuse alveolar hemorrhage (DAH) is a rare but life-threatening complication of SLE, and it presents clinically with hemoptysis, dyspnea, and anemia and radiographically with bilateral pulmonary infiltrates.

The clinical course of SLE is highly variable, often marked by unpredictable periods of flares and remissions. Although there is no universally accepted definition of a disease flare, it is generally understood among clinicians as a significant and measurable rise in disease activity that warrants a change in treatment [4].

## Case presentation

We describe the case of a 35-year-old woman with APS and SLE, who was receiving warfarin for a history of DVT and ischemic stroke. The patient presented to the emergency department with asthenia and productive cough with hemoptysis. Laboratory findings revealed hypochromic microcytic anemia, thrombocytopenia, and acute kidney injury. The international normalized ratio (INR) was within the target therapeutic range (Table 1).

**Table 1:** Laboratory parameters showing hypochromic microcytic anemia, thrombocytopenia, and acute kidney injury. INR: international normalized ratio

Parameters	Results	Reference values
Hemoglobin	6.9 g/dL	12.0-16.0 g/dL
Mean corpuscular hemoglobin	21.8 pg	27.0-35.0 pg
Mean corpuscular volume	75.4 fL	87.0-103.0 fL
Platelets	104 x 10^9^/L	150-400 x 10^9^/L
Albumin	34.1 g/L	38.0-51.0 g/L
Creatinine	2.07 mg/dL	0.51-0.95 mg/dL
Urea	94 mg/dL	10.00-50 mg/dL
INR	3.1	-
Summary urine test		
Protein	3.00 g/L	<0.15 g/L
Albumin	>150 mg/L	<30 mg/L

**Figure 1:**
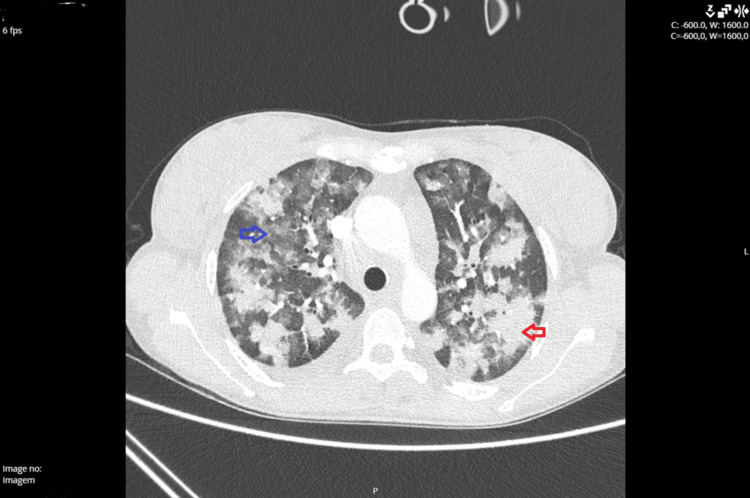
CT angiography showing extensive areas of consolidation (red arrow) and ground-glass densification (blue arrow) that raise the hypothesis of alveolar hemorrhage.

Imaging showed bilateral consolidation and ground-glass densification (Figure 1). Bronchoscopy was performed, with findings of intense neutrophilic alveolitis and positive Perls staining, compatible with mild alveolar hemorrhage.

She was transfused with one unit of red blood cell concentrate without complications. From the immunological study, positive ANAs > 1/1,000 with a homogeneous pattern; increased anti-DsDNA, anticardiolipins, and B2GP; and a positive lupus anticoagulant (LA) test should be highlighted (Table 2).

**Table 2:** The immunological study showed positivity for some antibodies.

Parameters	Results	Reference values
ANA (antinuclear antibodies)	>1/1,000, homogeneous pattern	<1/100
Anti-dsDNA	183.6 IU/mL	<100 IU/mL
Anticardiolipins		
IgG	280 GPL/mL	<20.0 GPL/mL
IgM	143 MPL/mL	<20.0 MPL/mL
β2-Glycoprotein		
IgG	222 U/mL	<20.0 U/mL
IgM	103 U/mL	<20.0 U/mL
Lupus anticoagulant (LA)	Positive	-

A diagnosis of lupus flare with pulmonary-renal syndrome was made, characterized by DAH and glomerulonephritis. Warfarin was suspended due to high bleeding risk - HAS-BLED score 3. Given the patient’s high thrombotic risk, anticoagulation with unfractionated heparin (UFH) was initiated 10 days later, due to clinical stabilization. However, she developed severe thrombocytopenia, prompting the suspension of anticoagulation. Heparin-induced thrombocytopenia (HIT) was excluded based on negative anti-PF4 antibodies. Other causes of thrombocytopenia were considered: Bactrim hematologic toxicity (replaced by atovaquone), autoimmune thrombocytopenia refractory to immunosuppression (corticosteroid dose was increased), and infection (hypothesis that was discarded).

As the platelet count recovered, UFH was restarted but led to a recurrence of DAH, necessitating another suspension of anticoagulation. While the patient was in intensive and intermediate care, anticoagulation was withheld due to high bleeding risk. Once stabilized and transferred to the ward, anticoagulation with low-molecular-weight heparin (LMWH) 1 mg/kg twice a day was introduced without recurrence of bleeding.

Despite clinical stability and having stopped bleeding, anemia persisted, which was interpreted as being multifactorial: hemolytic (positive direct Coombs test and haptoglobin consumption)/chronic disease/iron deficiency. Despite being scheduled, there were no conditions to safely perform a renal biopsy (hypocoagulation, thrombocytopenia) to rule out lupus nephritis; an outpatient nephrology consultation was scheduled.

It was a long hospital stay, with the need for glucocorticoids, cyclophosphamide, intravenous immunoglobulin (IVIG), and rituximab to manage the disease. She was treated with prednisolone 1 mg/kg/day, having undergone induction with cyclophosphamide; IVIG (1 g/kg/day) was also administered for four days due to refractory thrombocytopenia, and she completed two infusions of rituximab 1 g according to the protocol, without complications. At discharge, she was transitioned back to warfarin and started prednisolone 20 mg/day.

## Discussion

Managing anticoagulation in patients with concomitant APS and SLE presents a significant clinical challenge, especially when both thrombotic and hemorrhagic complications coexist. This case underscores the complexity of balancing the prevention of life-threatening thrombosis with the management of severe bleeding, such as DAH, in a patient with overlapping APS and active SLE.

APS is characterized by the presence of aPLs - including LA, anticardiolipin antibodies, and anti-β2-glycoprotein I antibodies - and is clinically defined by recurrent arterial or venous thromboses and/or pregnancy morbidity [5]. APS requires long-term anticoagulation, typically with vitamin K antagonists (VKAs) such as warfarin, to prevent recurrent thromboembolic events [6]. VKAs, particularly warfarin with a target INR of 2.0-3.0 (or even 3.0-4.0 in arterial events), remain the gold standard for secondary prophylaxis [7,8]. In our case, the patient had both a prior ischemic stroke and DVT, highlighting a significant thrombotic burden and necessitating sustained anticoagulation therapy.

The use of direct oral anticoagulants (DOACs) has been associated with a higher risk of thrombosis, particularly arterial thrombosis, compared with VKA. A recent randomized, open-label trial comparing rivaroxaban to warfarin in APS - Trial of Rivaroxaban in AntiPhospholipid Syndrome (TRAPS) - with triple aPL positivity (~21% of patients had SLE-APS) was prematurely terminated due to an excess of thromboembolic events in the rivaroxaban arm [9].

DAH is a rare but life-threatening complication of SLE, associated with high morbidity and mortality. It presents clinically with hemoptysis, dyspnea, and anemia and radiographically with bilateral pulmonary infiltrates [10]. Mortality rates are high, exceeding 50% in some series, particularly in cases where immunosuppressive therapy is delayed [11]. The pathogenesis is often related to capillaritis and immune complex deposition, necessitating prompt immunosuppressive therapy. The need to halt warfarin in this context reflects a critical management dilemma. While the interruption of anticoagulation increases the risk of recurrent thrombosis, its continuation may be fatal in the presence of active hemorrhage.

An initial strategy involved transitioning to UFH following clinical stabilization, a common approach given its short half-life and reversibility with protamine sulfate. This enables rapid modulation of anticoagulant effects in response to bleeding [12,13]. Unfortunately, our patient developed severe thrombocytopenia during heparin therapy. Despite negative anti-PF4 antibodies, which excluded HIT, thrombocytopenia necessitated the temporary suspension of anticoagulation.

This case also illustrates the importance of immunosuppressive therapy in addressing the underlying SLE activity. High-dose corticosteroids and cytotoxic immunosuppressants were initiated to control SLE activity. Cyclophosphamide, a potent immunosuppressant, is often employed in severe organ-threatening SLE manifestations such as DAH. Additionally, rituximab has shown efficacy in refractory cases due to its B-cell-depleting properties, aligning with emerging evidence supporting its use in severe lupus-related pulmonary involvement [14,15]. This case, together with other reports, suggests that rituximab might be an alternative drug for the treatment of DAH. Although frequently added to conventional therapies in refractory cases, the encouraging outcomes presented in several reports set forth the possibility of designing controlled studies to examine the efficacy of rituximab in the induction phase [16].

As the patient’s bleeding risk decreased and platelet counts recovered, anticoagulation was cautiously reintroduced with LMWH, transitioning later to warfarin with careful INR monitoring, without recurrence of hemorrhagic events. Recurrent APS-related events are common and difficult to predict. Therefore, the Global Antiphospholipid Score (GAPSS) was developed for the assessment of thrombosis risk in APS [17]. This tool can help us guide future cases. There are no specific bleeding scores for APS, and the Damage Index for Antiphospholipid Syndrome (DIAPS) does not include bleeding as damage. Further studies are needed in order to support the inclusion of damage caused by bleeding events in future revisions of the DIAPS [18].

This staged and individualized approach reflects current best practices in managing complex autoimmune conditions with competing risks. There is no standardized guideline for anticoagulation in patients with APS and concurrent major bleeding due to SLE, and therapeutic decisions must be guided by dynamic clinical assessment and multidisciplinary input. This case highlights the need for careful risk stratification, close monitoring, and flexibility in anticoagulation management in this high-risk patient population.

## Conclusions

The coexistence of APS and SLE poses significant therapeutic challenges, particularly when patients present with both thrombotic and hemorrhagic manifestations. This case illustrates the delicate balance required in managing anticoagulation in the context of active bleeding, emphasizing the importance of individualized treatment plans guided by clinical status, risk stratification, and multidisciplinary collaboration. Prompt recognition and aggressive immunosuppressive therapy are essential to control lupus flares and stabilize the patient, allowing for the safe reintroduction of anticoagulation. Ultimately, successful outcomes in such complex scenarios depend on continuous reassessment and tailored, dynamic management strategies. Further research and consensus guidelines are needed to optimize outcomes in this challenging subgroup. This patient is currently medicated with warfarin and is clinically stable, with no new thrombotic or hemorrhagic episodes.
